# Beyond the Tragedy: Illuminating Challenges in Disaster Management and Mental Health Support in Resource-Constrained Environments

**DOI:** 10.1017/S1049023X24000657

**Published:** 2024-12

**Authors:** Syed Muhammad Aqeel Abidi

**Affiliations:** Medicine and Surgery, The Aga Khan University, Karachi, Pakistan

**Keywords:** community resilience, disaster management, disaster preparedness, humanitarian efforts, mental health support, Pakistan flooding, public health

## Abstract

In the aftermath of the 2022 Pakistan flooding, disaster management faced critical challenges, particularly in mental health support. This study analyzed an incident where eighteen internally displaced individuals lost their lives in a bus fire. The current approach involves a comprehensive analysis of the incident, exploring the difficulties encountered in managing relief efforts, and providing mental health support. The study aims were to evaluate existing mental health support mechanisms, to identify challenges in disaster management, and to propose recommendations for future preparedness. Recommendations include enhancing disaster response training, integrating mental health services into primary health care, and prioritizing community resilience. These insights contribute to a deeper understanding of disaster management in resource-constrained regions.

## Specific Event Identifiers

- **Event Type:** Bus Fire Incident – During the return journey of flood victims
- **Event Onset Date:** October 12, 2022
- **Location of Event:** Nooriabad, Sindh, Pakistan
- **Geographic Coordinates:** 25.2740° N, 68.4870° E
- **Date(s) of Observations Reported:** October 12, 2022
- **Response Type:** Humanitarian Assistance, Medical Relief

## Introduction

In 2022, Pakistan faced a catastrophic natural disaster of unprecedented scale as the nation grappled with extensive flooding. Among the regions profoundly affected by this was Khairpur Nathanshah, a rural area in the middle of the Sindh province. This area is next to the river Indus and comprises mostly farmlands. Most of the population lives in poor living conditions due to limited resources and infrastructure. The inundation of this region left communities devastated, their lives upturned, and their futures uncertain.

Faced with the daunting challenge of rebuilding amidst the deluge, many displaced individuals sought refuge in the city of Karachi, finding temporary shelter in Internally Displaced Persons (IDP) camps. Amid this dire crisis, multiple humanitarian organizations mobilized resources to extend a helping hand. The efforts encompassed a holistic approach, encompassing the provision of health care services, the distribution of essential supplies such as food, clothing, and hygiene items, and the establishment of medical camps to address the immediate health needs of the affected individuals. These endeavors represented a beacon of hope for the flood-affected communities, offering vital support and assistance during a profoundly challenging period. However, amidst the backdrop of this extensive relief effort, an event unfolded that would leave a permanent mark on the hearts and minds of those involved.

On the journey back to their homes after months of displacement, a bus carrying twelve different families, belonging to the same extended family, from the IDP camps in Karachi, which had served as their temporary refuge, met a devastating fate. The bus, which was reported to have around forty-five to forty-seven passengers on board, tragically caught fire, and in the ensuing inferno, eighteen people, twelve of whom were children and five women, were consumed by the flames.

No detailed investigation was carried out into the incident, but the preliminary reports suggested that this was due to a short circuit in the air-conditioning system of the bus. There was a sudden breakout of the fire from the rear of the bus, which didn’t give the passengers enough time to react. And as there were steel bars on the windows of the bus, the people who were seated towards the back could not escape the fire. The severity of the fire was such that relatives could not recognize the bodies of their deceased peoples. The incident was a profound tragedy, not only in terms of the lives lost, but also for its emotional and psychological toll on survivors, witnesses, and those who had been diligently working to provide aid and support.

This article examines the significant impact of a distressing event during flood relief efforts, emphasizing the challenges in resource-constrained settings. It underscores the crucial role of disaster management and mental health support. The objective is to comprehensively explore the incident’s implications, drawing lessons to inform future disaster response and mental health support in similar contexts, especially in low- and middle-income countries (LMICs). This article aims to contribute to a deeper understanding of the intersection between disaster management, public health, and community resilience.

## Sources

Humanity Initiative (HI; Pakistan) nongovernmental organization (NGO)’s president was coordinating flood relief efforts with multiple NGOs committed to alleviating the suffering inflicted by the floods. In this IDP camp, the president was personally involved in looking after the health needs of the people and helped in managing for the ration for the people.

After this incident, the president was directly in contact with the head of the IDP camp who belonged to Khairpur Nathanshah and coordinated all the relief efforts in the city. He kept HI’s president updated with all the happenings with the survivors and long-term outcome. Additionally, information was gathered through multiple different news outlets, as well as conversing with government officials and looking at the official reports of the incident released.^1–3^

## Observation

The affected population in Khairpur Nathanshah found themselves amid a calamity of immense proportions during 2022. First, they experienced the full force of nature’s fury as floodwaters inundated their homes and farmlands. And then, inside the IDP camps of Karachi, in unfamiliar surroundings and away from their homes and communities, they confronted the challenges of displacement, uncertainty, and vulnerability. These Internally Displaced refugees were finally heading back, after months of being away from home, dreaming of sleeping in their warm beds and rebuilding their lives. The organizers were similarily elated, as they worked hard for months to reach this moment.

However, in a horrifying turn of events, the bus carrying these families became engulfed in flames, leading to a devastating loss of life. The immediate aftermath of the incident was marked by scenes of unimaginable horror and grief, with survivors and witnesses grappling with shock and despair. These victims all belonged to a single extended family. When HI’s president contacted the supervisor of relief operations in Khairpur Nathanshah, he explained that there was intense grief in the family. Those who were present in the bus were struggling with dealing with the memory of the event and many had symptoms of posttraumatic stress disorder/PTSD (ie, nightmares and insomnia), and those who weren’t on the bus were in grief due to losing so many relatives in such a sudden accident. However, there was no psychological support extended to the family, and as Khairpur Nathanshah is a rural city, the line of contact with the survivors was severed as well.

While the incident’s consequences rippled through the affected communities, it also had a profound emotional and psychological effect on the relief workers who had been tirelessly supporting them. They had been providing help and assistance to these families, and now were grappling with a sense of helplessness in the face of such a tragedy. This incident highlighted that while generally mental health and psychosocial support are administered to the survivors and witnesses of such traumatic experiences, the relief workers are neglected at such times. For their efficient functioning, it is necessary. However, in this case, neither the survivors nor the caregivers received any support.

For months following the incident, many responders grappled with the emotional aftermath of the tragedy. Interacting closely with IDPs created lasting emotional impressions, with many experiencing recurring flashbacks and emotional distress. Despite efforts to maintain emotional resilience, not all responders were able to visit the site of the fire or confront the full details of the event. While some were able to seek professional help from psychiatrists or psychologists, the process of recovery was gradual.

A multitude of factors contributed to this tragic incident, and it brought to light the complexities of disaster management in resource-constrained settings. The incident exposed limitations in pre-disaster planning and response strategies. The sheer scale and suddenness of the flooding had already exhausted existing disaster management protocols, and the lack of a central response body meant the efforts were mostly individualized and not coordinated. It became evident that a more comprehensive approach to disaster preparedness and response was needed, one that accounted for the unique challenges posed by the region’s vulnerability to natural disasters.

The lack of a coordinated central response to the disaster meant that organizations were not aware of the time of the journey of the IDPs. The bus that was arranged was a private bus that usually runs inter-city in Pakistan; this journey was not arranged by the volunteers, rather it was the arrangement of the IDPs themselves. As this was just one extended family, they unilaterally decided that they had to return to their city to try and recover from the situation. As a result, issues related to bus safety measures were overlooked. One can make the speculation that issues like maintenance, emergency exits, and fire safety equipment were not looked into as they wanted the cheapest option to travel.

Most of the deceased were children; this could be because in such emergency situations, it is difficult for children to protect themselves. One of the morgue officials had stated that: “The body of one woman was recovered from the bus who was holding her little child in her lap. Perhaps she wanted to save her child and both were burnt to death. Their bodies were stuck together in death and buried together.”^4^

## Analysis

The incident underscores the requirement of strong mental health support mechanisms in disaster-prone, resource-constrained settings. Evaluating the existing mental health support systems in such contexts reveals significant gaps and inadequacies. Limited access to mental health professionals, stigma associated with seeking psychological assistance, long distances to rural areas, lack of infrastructure in rural/disaster-affected areas, and a shortage of resources for comprehensive mental health care are among the prominent challenges.^5^

Providing adequate mental health services in LMICs, particularly in the aftermath of a disaster, presents a complex set of hurdles. The incident shed light on the need for innovative approaches, such as task-shifting to community health workers and the integration of mental health services into primary health care systems. These measures are crucial in extending the reach of mental health care to the most vulnerable populations in resource-constrained settings.

Furthermore, the incident emphasized the paramount importance of psychosocial support in disaster relief efforts. Beyond clinical interventions, the provision of psychosocial support, which encompasses emotional and social assistance, is central to helping survivors and relief workers cope with the trauma and grief they experienced.^6^ This comprehensive approach recognizes that healing in the aftermath of a disaster extends beyond the patient’s physical state and highlights the significance of addressing emotional and psychological well-being. However, being an LMIC and in a state of emergency, no such intervention was provided.

The incident served as a stark reminder of the critical need to prioritize mental health support in disaster-prone, resource-constrained environments. It underscored the urgency of developing tailored mental health strategies that account for the unique challenges and circumstances faced by communities in LMICs grappling with the aftermath of natural disasters. Mental health must be recognized as an integral component of disaster relief efforts, as survivors, witnesses, and relief workers all grapple with the psychological aftermath of traumatic events.^7^ The incident serves as a compelling case study demonstrating that mental health support is not an ancillary concern, but a central pillar of resilience and recovery in disaster-prone regions.

To address this, recommendations emerge for enhancing disaster management and mental health support in LMICs. These include regular mental health screening for relief workers, expanding access to mental health services, reducing stigma surrounding mental health, and promoting community-based psychosocial support systems.^8^ By heeding these recommendations, responders can strive to ensure that future disaster response efforts are not only more effective, but also more compassionate, recognizing and addressing the mental and emotional needs of affected communities in resource-constrained environments.

In the aftermath of the tragic bus fire incident during the relief efforts amidst the 2022 Pakistan flooding, researchers find themselves confronted with a somber reminder of the multi-layered challenges that disaster management and mental health support entail in resource-constrained environments. This heart-wrenching event, which claimed the lives of twelve families returning to their homes after months of displacement, stands as a symbol of the trials faced by communities in the wake of natural disasters.
